# Diaphorin, a polyketide produced by a bacterial endosymbiont of the Asian citrus psyllid, adversely affects the *in vitro* gene expression with ribosomes from *Escherichia coli* and *Bacillus subtilis*

**DOI:** 10.1371/journal.pone.0294360

**Published:** 2023-11-14

**Authors:** Rena Takasu, Yuka Yasuda, Takashi Izu, Atsushi Nakabachi

**Affiliations:** 1 Department of Applied Chemistry and Life Science, Toyohashi University of Technology, Toyohashi, Aichi, Japan; 2 Research Center for Agrotechnology and Biotechnology, Toyohashi University of Technology, Toyohashi, Aichi, Japan; Boyce Thompson Institute, UNITED STATES

## Abstract

Diaphorin is a polyketide produced by “*Candidatus* Profftella armatura” (Gammaproteobacteria), an obligate mutualist of an important agricultural pest, the Asian citrus psyllid *Diaphorina citri* (Hemiptera). Our previous study demonstrated that diaphorin, at physiological concentrations in *D*. *citri*, inhibits the growth and cell division of *Bacillus subtilis* (Firmicutes) but promotes the growth and metabolic activity of *Escherichia coli* (Gammaproteobacteria). This unique property of diaphorin may aid microbial mutualism in *D*. *citri*, potentially affecting the transmission of “*Candidatus* Liberibacter spp.” (Alphaproteobacteria), the pathogens of the most destructive citrus disease Huanglongbing. Moreover, this property may be exploited to promote microbes’ efficiency in producing industrial materials. However, the mechanism underlying this activity is unknown. Diaphorin belongs to the family of pederin-type compounds, which inhibit protein synthesis in eukaryotes by binding to eukaryotic ribosomes. Therefore, as a first step to assess diaphorin’s direct influence on bacterial gene expression, this study examined the effect of diaphorin on the *in vitro* translation using ribosomes of *B*. *subtilis* and *E*. *coli*, quantifying the production of the green fluorescent protein. The results showed that the gene expression involving *B*. *subtilis* and *E*. *coli* ribosomes along with five millimolar diaphorin was 29.6% and 13.1%, respectively, less active than the control. This suggests that the diaphorin’s adverse effects on *B*. *subtilis* are attributed to, at least partly, its inhibitory effects on gene expression. Moreover, as ingredients of the translation system were common other than ribosomes, the greater inhibitory effects observed with the *B*. *subtilis* ribosome imply that the ribosome is among the potential targets of diaphorin. On the other hand, the results also imply that diaphorin’s positive effects on *E*. *coli* are due to targets other than the core machinery of transcription and translation. This study demonstrated for the first time that a pederin congener affects bacterial gene expression.

## Introduction

Microbes produce various secondary metabolites mediating interactions with neighboring organisms [1, 2]. These molecules exhibit diverse biological activities, some of which facilitate symbiotic relationships between microbes and animal hosts [3–5].

Diaphorin is a polyketide produced by “*Candidatus* Profftella armatura” (Gammaproteobacteria: Burkholderiales), an organelle-like obligate symbiont harbored along with the primary symbiont “*Ca*. Carsonella ruddii” (Gammaproteobacteria: Oceanospirillales) [6, 7] in the bacteriome organ [8, 9] of the Asian citrus psyllid *Diaphorina citri* (Hemiptera: Psyllidae) [10–13]. *D*. *citri* is a major agricultural pest that transmits “*Candidatus* Liberibacter spp.” (Alphaproteobacteria: Rhizobiales), the pathogens of the most destructive and incurable citrus disease called Huanglongbing or greening disease [14, 15]. Conservation of *Profftella* and its gene clusters for diaphorin synthesis in *Diaphorina* spp. highlights the physiological and ecological significance of diaphorin for the host psyllids [16, 17]. Previous studies indicated that diaphorin, which exists at a concentration as high as 2–20 mM in *D*. *citri* [18], exerts inhibitory effects on various eukaryotic organisms, including the natural enemies of *D*. *citri* [10, 19, 20]. A recent study further revealed that physiological concentrations of diaphorin also suppress the growth and cell division of the model bacterium *Bacillus subtilis* (Firmicutes: Bacilli) but promote the growth and metabolic activity of another model bacterium *Escherichia coli* (Gammaproteobacteria: Enterobacterales) [21]. These findings suggest that diaphorin serves as a defensive agent of the holobiont (host + symbionts) against eukaryotes [10, 19, 20] and some bacterial lineages but is metabolically beneficial for other bacteria, which potentially include obligate symbionts of *Diaphorina* spp. and plant pathogens that they transmit [21]. Furthermore, since *E*. *coli* is utilized for producing various industrially important materials, including pharmaceutical drugs, amino acids, enzymes, and biofuels [22], the observed effects of diaphorin could potentially be exploited to enhance the efficiency of industrial material production by *E*. *coli* [21]. Nonetheless, the mechanism underlying this unique biological activity remains unknown.

Diaphorin belongs to the family of pederin-type compounds observed in a variety of host-symbiont systems, including beetles, lichens, and sponges, which are home to diverse bacterial producers [10, 21]. These pederin congeners display toxicity and antitumor activity by binding to the E-site of the 60S subunit of eukaryotic ribosomes and suppressing eukaryotic protein synthesis [23–25]. However, little is known about the effects of these compounds on bacterial gene expression, as they were previously thought to have minimal impact on bacteria [26, 27]. Therefore, as a first step to explore the possibility that diaphorin exerts its distinct activity in bacteria by directly targeting bacterial gene expression, the present study investigated the effects of diaphorin on the *in vitro* gene expression using ribosomes isolated from *B*. *subtilis* and *E*. *coli*, quantifying cell-free production of the super folder green fluorescent protein.

## Materials and methods

### Preparation of diaphorin

Diaphorin was extracted and purified as described previously [10, 19, 21]. Adult *D*. *citri* were ground in methanol, and the extracts were concentrated *in vacuo*. The residue was purified using an LC10 high-performance liquid chromatography system (Shimadzu) with an Inertsil ODS-3 C18 reverse-phase preparative column (GL Science). The purified samples were combined, dried, redissolved in methanol, and filtered through a Minisart syringe filter with a pore size of 0.2 μm (Sartorius). Aliquots of the purified samples were quantified in the LC10 HPLC system using an Inertsil ODS-3 analytical column (GL Science). The purified diaphorin was stored at −20°C until use.

### Preparation of DNA templates for the *in vitro* synthesis of sfGFP

The DNA template for cell-free synthesis of sfGFP [28] was prepared using PCR (S1 Fig). First PCR to amplify the *sfGFP* gene, introducing a ribosome binding site upstream, was performed with KOD -Plus- Neo (TOYOBO) and the primer set GFP_purefrex_F (5′- AAGGAGATATACCAATGAGTAAAGGAGAAGAACTTTTCAC-3′, underline: ribosome binding site) and GFP_purefrex_R2 (5′-GGATTAGTTATTCATTATTTGTAGAGCTCATCCATGCC-3′), using pRP[Exp]-CMV>sfGFP plasmid (VectorBuilder) as a template. Running parameters were 94°C for 2 min, followed by 30 cycles of 98°C for 10 s, 58°C for 30 s, and 68°C for 30 s. The second PCR to introduce the T7 promoter upstream of the ribosome binding site was performed with KOD -Plus- Neo and the primer set T7PRO_SD (5′- GAAATTAATACGACTCACTATAGGGAGACCACAACGGTTTCCCTCTAGAAATAATTTTGTTTAACTTTAAGAAGGAGATATACCA-3′, underline: T7 promoter) and GFP_purefrex_R2, using a 1/100 dilution of the first PCR product as a template. The running parameters were the same as in the first PCR. The obtained product was purified with a FastGene Gel/PCR extraction kit (NIPPON Genetics) following the manufacturer’s instructions. The nucleotide sequence of the PCR product was checked by Sanger sequencing.

### Preparation of the *Bacillus subtilis* ribosome

Ribosomes from *B*. *subtilis* were purified according to a previous report [29] with some modifications. *B*. *subtilis* strain ISW1214 transformed with the pHY300PLK plasmid [21] was cultured in L-broth (1% Bacto tryptone, 0.5% Bacto yeast extract, 0.05% NaCl, pH 7.0) containing 20 μg/mL tetracycline at 30°C for 20 h with reciprocal shaking (130 rpm). Cells were harvested by centrifugation (3,000 ×g, 4°C, 5 min), washed twice with buffer I-H [10 mM 4-(2-hydroxyethyl)-1-piperazineethanesulfonic acid (HEPES)-KOH pH 7.6, 15 mM Mg(AOc)_2_, 1 M KCl, 5 mM ethylenediaminetetraacetic acid, 10 mM *β*-mercaptoethanol], then once with buffer II-H (Buffer I-H with 50 mM KCl) and stored as a pellet at −80°C. Cells were thawed in suspension buffer [10 mM HEPES-KOH pH 7.6, 10 mM Mg(OAc)_2_, 50 mM KCl, 7 mM *β*-mercaptoethanol, 2 U/mL RNase-free DNase I (Nippon Gene), 8 U/mL recombinant RNase inhibitor (Takara), 1 mg/mL Pefabloc SC (Merck Millipore), 10 μg/mL Leupeptin (FUJIFILM Wako), 0.7 μg/mL Pepstatin (FUJIFILM Wako)] and passed through a precooled French press cell (Ohtake) at approximately 110 MPa (16,000 psi). The cell lysate was mixed with an equal volume of suspension buffer with 3 M ammonium sulfate and kept on ice for 30 min. After cell debris and protein precipitates were removed by centrifugation (10,000 ×g, 4°C, 30 min), the supernatant was subjected to HiTrap Butyl FF columns (Cytiva) equilibrated with buffer A (20 mM HEPES-KOH pH 7.6, 1.5 M (NH4)_2_SO_4_, 10 mM Mg(OAc)_2_, 7 mM *β*-mercaptoethanol). The column was then washed with 40 mL of buffer B (20 mM HEPES-KOH pH 7.6, 10 mM Mg(OAc)_2_, 7 mM *β*-mercaptoethanol) containing 1.2 M ammonium sulfate, and proteins were eluted with a 1.2 M–0 M gradient of ammonium sulfate in buffer B. The abundance of ribosomes eluted in each fraction was determined by measuring absorbance at 260 nm using a NanoDrop 2000c spectrophotometer (Thermo Fisher Scientific). The combined fractions containing the ribosome were loaded on top of an equal volume of 30% sucrose cushion buffer (20 mM HEPES-KOH pH 7.6, 10 mM Mg(OAc)_2_, 30 mM NH_4_Cl, 30% sucrose, 7 mM *β*-mercaptoethanol) and ultracentrifuged (100,000 ×g, 4°C, 16 h) using Optima L-100 XP Ultracentrifuge (Beckman Coulter) to sediment the ribosomes. The pellet was suspended in ribosome buffer (20 mM HEPES-KOH pH 7.6, 30 mM KCl, 6 mM Mg(OAc)_2_, 7 mM *β*-mercaptoethanol) and stored at −80°C until use.

### Cell-free synthesis of sfGFP

The *in vitro* gene expression activities using ribosomes derived from *E*. *coli* and *B*. *subtilis* were evaluated utilizing a PURE*frex 2*.*0* kit (GeneFrontier). PURE*frex* is a cell-free protein synthesis system reconstructed using purified substrates and proteins related to transcription and translation that have been individually purified to homogeneity [30]. To determine the gene expression activity involving the *E*. *coli* ribosome, sfGFP was synthesized following the manufacturer’s instruction using nuclease-free water, the solution I (amino acids, NTPs, creatine phosphate, 10-formyl-5,6,7,8-tetrahydrofolic acid, tRNAs), solution II (IF1, IF2, IF3, EF-G, EF-Tu, EFTs, RF1, RF3, RRF, aminoacyl-tRNA synthetases, methionyl-tRNA transformylase, T7 RNA polymerase, nucleoside diphosphate kinase, myokinase, creatine kinase, pyrophosphatase), solution III (the *E*. *coli* ribosome), and template DNA prepared as above. Protein synthesis was performed at 37°C for 4 hours. To evaluate the gene expression activity involving the *B*. *subtilis* ribosome, sfGFP was synthesized following the same procedure as described, except that solution III was replaced with the ribosomes purified from *B*. *subtilis* as described above. Previous studies have demonstrated that the *B*. *subtilis* ribosome can effectively synthesize proteins with the gene expression machinery derived from *E*. *coli* [29].

### Quantification of synthesized sfGFP

Reaction solutions of cell-free translation of sfGFP were mixed with two volumes of water and an equal volume of 4×sample buffer [200 mM Tris-HCl pH 6.8, 40% glycerol, 8% sodium dodecyl sulfate (SDS), 24% *β*-mercaptoethanol, 0.01% bromophenol blue], and incubated at 80°C for 5 min. The translation products of four samples each from the control and experimental groups were loaded on a 14% conventional Laemmli gel and separated along with the size marker Precision Plus Protein Dual Color Standards (Bio-Rad) by SDS-polyacrylamide gel electrophoresis at 200 V constant voltage for 70 minutes. Subsequently, gels were shaken for 4 hours at room temperature in the Western blotting transfer buffer (25 mM Tris, 192 mM glycine, 20% methanol) to renature sfGFP. The fluorescence of sfGFP was elicited at 488 nm, passed through a 520 nm band pass filter, and recorded using a Typhoon 9400 image analyzer (GE Healthcare). Size marker proteins were visualized with a 633 nm laser and 670 nm band pass filter to check the size of sfGFP (approximately 27 kDa). The fluorescence intensity of sfGFP was quantified using the Analysis Toolbox module of ImageQuant TL software (version 8.1, GE Healthcare). Areas for sfGFP bands were defined using the rectangle tool to quantify the signal intensity. The baseline signal was established for each gel, and the signal intensity of each sample was subsequently adjusted by subtracting the baseline value. The average value of four control samples was calculated and used to normalize the signal intensity values of all samples loaded on the same gel to determine their relative values. The analysis of diaphorin treatment was based on data from 48 independent translation reactions for each group, which were loaded and separated on 12 gels. The analysis of tetracycline treatment was based on data from 12 independent translation reactions for each group, which were loaded and separated on three gels. Preliminary analysis indicated that the presence of diaphorin does not directly affect the fluorescence of the coexisting GFPs.

### Statistical analysis

All statistical analyses were conducted using R version 4.1.3 [31]. Kolmogorov–Smirnov and Shapiro–Wilk tests [32, 33] were used to determine whether the data were normally distributed. If the normal distribution was rejected, data from the two groups were compared using the Brunner–Munzel test, a nonparametric method that does not rely on homoscedasticity [34]. The Bartlett test [35] was used to assess the homogeneity of variances for multiple comparisons. If the normal distribution or homogeneous data variance was rejected, multiple comparisons were conducted using the Kruskal-Wallis test [36]. Post hoc analysis following the Kruskal-Wallis test was performed by the Steel-Dwass test [37].

## Results and discussion

**Fig 1:**
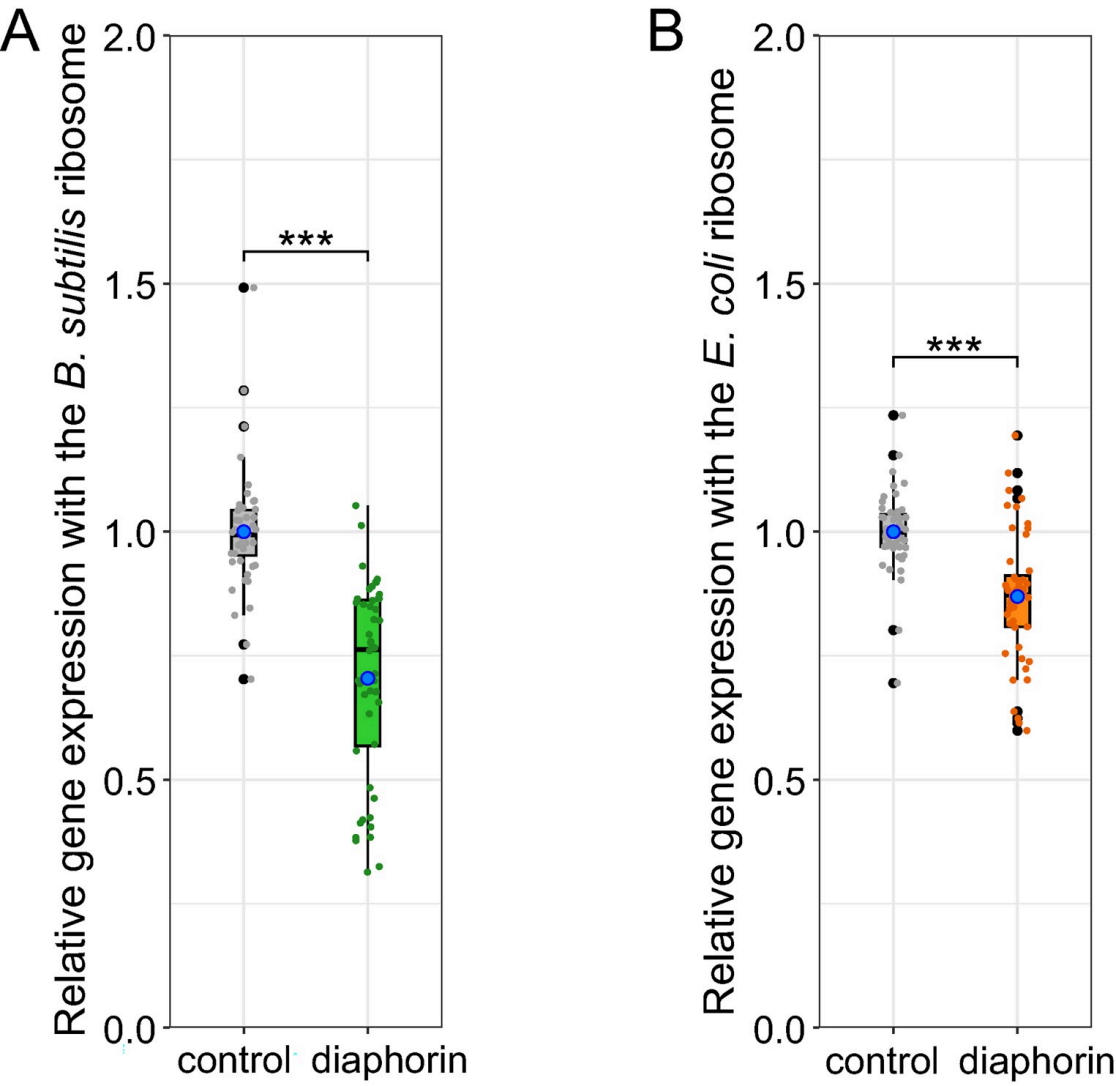
Effects of diaphorin on bacterial gene expression *in vitro*. (A) Relative gene expression with the *B*. *subtilis* ribosome. The signal intensity of synthesized sfGFP in each sample is normalized to the mean signal intensity of control samples. Jitter plots of all data points (*n* = 48) and box plots (gray, control; green, 5 mM diaphorin) showing their distributions (median, quartiles, minimum, and maximum) are indicated. Blue dots represent the mean. Asterisks indicate a statistically significant difference (***, *p* < 0.001, Brunner–Munzel test). (B) Relative gene expression with the *E*. *coli* ribosome. The signal intensity of synthesized sfGFP in each sample is normalized to the mean signal intensity of control samples. Jitter plots of all data points (*n* = 48) and box plots (gray, control; orange, 5 mM diaphorin) showing their distributions (median, quartiles, minimum, and maximum) are indicated. Blue dots represent the mean. Asterisks indicate a statistically significant difference (***, *p* < 0.001, Brunner–Munzel test).

Cell-free translation of sfGFP (S1 Fig) with or without diaphorin at a final concentration of 5 mM demonstrated that the physiological concentration of diaphorin adversely affects the *in vitro* gene expression involving ribosomes of both *B*. *subtilis* and *E*. *coli* (Fig 1). Namely, the relative gene expression activity using the *B*. *subtilis* ribosome along with 5 mM diaphorin was 0.704 ± 0.028 (mean ± standard error, *n* = 48), which was significantly (*p* < 0.001, Brunner–Munzel test) and considerably (29.6%) lower than that of the control (1.000 ± 0.017, *n* = 48, Fig 1A). This implies that the detrimental effects of diaphorin on the growth and cell division of *B*. *subtilis* can be attributed, at least in part, to its direct inhibitory effects on gene expression of *B*. *subtilis*. The relative activity of gene expression using the *E*. *coli* ribosome treated with 5 mM diaphorin was 0.869 ± 0.019 (*n* = 48), which was moderately (13.1%) but significantly (*p* < 0.001, Brunner–Munzel test) lower than that of the control (1.000 ± 0.012, *n* = 48, Fig 1B). This result is apparently inconsistent with the positive effects of the same concentration of diaphorin on the growth and metabolic activity of *E*. *coli* observed in the previous study [21], implying that the positive impact of diaphorin on *E*. *coli* cells is likely due to targets other than the core framework of gene expression system. This does not exclude the possibility that factors absent from the core gene expression machinery used in the present study (other than amino acids, NTPs, creatine phosphate, 10-formyl-5,6,7,8-tetrahydrofolic acid, tRNAs, IF1, IF2, IF3, EF-G, EF-Tu, EFTs, RF1, RF3, RRF, aminoacyl-tRNA synthetases, methionyl-tRNA transformylase, T7 RNA polymerase, nucleoside diphosphate kinase, myokinase, creatine kinase, pyrophosphatase, and ribosome) change the diaphorin’s effects on gene expression. Because ingredients of the translation system were common other than the ribosomes derived from two bacterial species, the greater degree of inhibition observed with the *B*. *subtilis* ribosome (*p* < 0.001, Steel-Dwass test) implies that the ribosome is among the potential targets of diaphorin. Further studies are required to identify the specific targets of diaphorin.

For reference, the effects of tetracycline, an antibiotic that specifically inhibits protein synthesis in bacteria by binding to the 30S ribosomal subunit [38], were assessed using the same *in vitro* translation system (S2 Fig). In the reaction involving the *B*. *subtilis* ribosome, the addition of 50 μM tetracycline reduced the relative gene expression activity to 0.353 ± 0.121 (mean ± standard error, *n* = 12), which was significantly (*p* < 0.001, Steel-Dwass test) and notably (64.7%) lower than that of the control (1.000 ± 0.042, *n* = 12, S2A Fig). Furthermore, 500 μM tetracycline reduced the relative gene expression activity to 0.110 ± 0.121 (*n* = 12), which was significantly (*p* < 0.001, Steel-Dwass test) lower than that of the control and 50 μM tetracycline-treated group (S2A Fig). In the reaction involving the *E*. *coli* ribosome, 50 μM tetracycline reduced the relative gene expression activity to 0.539 ± 0.013 (*n* = 12), which was significantly (*p* < 0.001, Steel-Dwass test) and considerably (46.1%) lower than that of the control (1.000 ± 0.034, *n* = 12, S2B Fig). Moreover, 500 μM tetracycline reduced the relative translation activity to 0.122 ± 0.011 (*n* = 12), which was significantly (*p* < 0.001, Steel-Dwass test) lower than that of the control and 50 μM tetracycline-treated group (S2B Fig). These results corroborated that the *in vitro* translation system used in the present study is suitable for assessing the impacts of substances on the activity of the core framework of bacterial gene expression. In future studies, we will be able to use this system to assess the influence of candidate targets of diaphorin, which are identified by techniques including affinity assays and multiomics analyses.

The present study demonstrated for the first time that a pederin-type compound affects bacterial gene expression, shedding light on the mechanism for diaphorin’s distinctive activity. Furthermore, this study highlights the potential of the pederin family compounds, motivating further research on their influence and activities on bacteria.

## Supporting information

S1 Fig Nucleotide (a part of the pRP[Exp]-CMV>sfGFP plasmid) and amino acid sequences of the sfGFP (yellow shading) and primers for PCRs to prepare the template DNA for cell-free translation. T7 promoter and ribosome binding site are indicated in blue and red, respectively.
(PDF)

S2 Fig Effects of tetracycline on bacterial gene expression *in vitro*. (A) Relative gene expression with the *B*. *subtilis* ribosome. The signal intensity of synthesized sfGFP in each sample is normalized to the mean signal intensity of control samples. Jitter plots of all data points (*n* = 12) and box plots (gray, control; green, 50 μM or 500 μM tetracycline) showing their distributions (median, quartiles, minimum, and maximum) are indicated. Blue dots represent the mean. Asterisks indicate a statistically significant difference (***, *p* < 0.001, Steel-Dwass test). (B) Relative gene expression with the *E*. *coli* ribosome. The signal intensity of synthesized sfGFP in each sample is normalized to the mean signal intensity of control samples. Jitter plots of all data points (*n* = 12) and box plots (gray, control; orange, 50 μM or 500 μM tetracycline) showing their distributions (median, quartiles, minimum, and maximum) are indicated. Blue dots represent the mean. Asterisks indicate a statistically significant difference (***, *p* < 0.001, Steel-Dwass test).
(PDF)
