# Supplementary material for: Diaphorin, a polyketide produced by a bacterial endosymbiont of the Asian citrus psyllid, adversely affects the in vitro gene expression with ribosomes from Escherichia coli and Bacillus subtilis
Source: PLoS One. 2023 Nov 14;18(11):e0294360. doi: 10.1371/journal.pone.0294360 (PMC10645341; doi:10.1371/journal.pone.0294360)
Supplement: S1 Fig — T7 promoter and ribosome binding site are indicated in blue and red, respectively. (PDF) [file pone.0294360.s001.pdf]

```
T7PRO_SD GAAATTAATACGACTCACTATAGGGAGACCACAACGGTTTCCC
          TCTAGAAATAATTTTGTTTAACTTTAAGAAGGAGATATACCA
                         GFP_purefrex_F AAGGAGATATACCAATGAGTAAAGGAGAAGAACT
           ...CGTCAGATCCAAGTTTGTACAAAAAAGCAGGCTGCCACCATGAGCAAAGGAGAAGAACT
           TTTCAC                                    M  S  K  G  E  E  L
           TTTCACTGGAGTTGTCCCAATTCTTGTTGAATTAGATGGTGATGTTAATGGGCACAAATT
            F  T  G  V  V  P  I  L  V  E  L  D  G  D  V  N  G  H  K  F
           TTCTGTCCGTGGAGAGGGTGAAGGTGATGCTACAAACGGAAAACTCACCCTTAAATTTAT
            S  V  R  G  E  G  E  G  D  A  T  N  G  K  L  T  L  K  F  I
           TTGCACTACTGGAAAACTACCTGTTCCGTGGCCAACACTTGTCACTACTCTGACCTATGG
            C  T  T  G  K  L  P  V  P  W  P  T  L  V  T  T  L  T  Y  G
           TGTTCAATGCTTTTCCCGTTATCCGGATCACATGAAACGGCATGACTTTTTCAAGAGTGC
            V  Q  C  F  S  R  Y  P  D  H  M  K  R  H  D  F  F  K  S  A
           CATGCCCGAAGGTTATGTACAGGAACGCACTATATCTTTCAAAGATGACGGGACCTACAA
            M  P  E  G  Y  V  Q  E  R  T  I  S  F  K  D  D  G  T  Y  K
           GACGCGTGCTGAAGTCAAGTTTGAAGGTGATACCCTTGTTAATCGTATCGAGTTAAAGGG
            T  R  A  E  V  K  F  E  G  D  T  L  V  N  R  I  E  L  K  G
           TATTGATTTTAAAGAAGATGGAAACATTCTTGGACACAAACTCGAGTACAACTTTAACTC
            I  D  F  K  E  D  G  N  I  L  G  H  K  L  E  Y  N  F  N  S
           ACACAATGTATACATCACGGCAGACAAACAAAAGAATGGAATCAAAGCTAACTTCAAAAT
            H  N  V  Y  I  T  A  D  K  Q  K  N  G  I  K  A  N  F  K  I
           TCGCCACAACGTTGAAGATGGTTCCGTTCAACTAGCAGACCATTATCAACAAAATACTCC
            R  H  N  V  E  D  G  S  V  Q  L  A  D  H  Y  Q  Q  N  T  P
           AATTGGCGATGGCCCTGTCCTTTTACCAGACAACCATTACCTGTCGACACAATCTGTCCT
            I  G  D  G  P  V  L  L  P  D  N  H  Y  L  S  T  Q  S  V  L
           TTCGAAAGATCCCAACGAAAAGCGTGACCACATGGTCCTTCTTGAGTTTGTAACTGCTGC
            S  K  D  P  N  E  K  R  D  H  M  V  L  L  E  F  V  T  A  A
                       CCGTACCTACTCGAGATGTTTATTACTTATTGATTAGG GFP_purefrex_R2
           TGGGATTACACATGGCATGGATGAGCTCTACAAATAAACCCAGCTTTCTTGTACAAAG...
            G  I  T  H  G  M  D  E  L  Y  K
```
