# Supplementary figures and images for: Diaphorin, a polyketide produced by a bacterial endosymbiont of the Asian citrus psyllid, adversely affects the in vitro gene expression with ribosomes from Escherichia coli and Bacillus subtilis
Source: PLoS One. 2023 Nov 14;18(11):e0294360. doi: 10.1371/journal.pone.0294360 (PMC10645341; doi:10.1371/journal.pone.0294360)

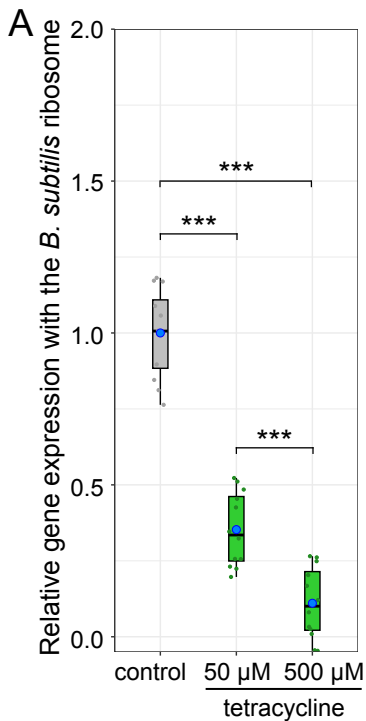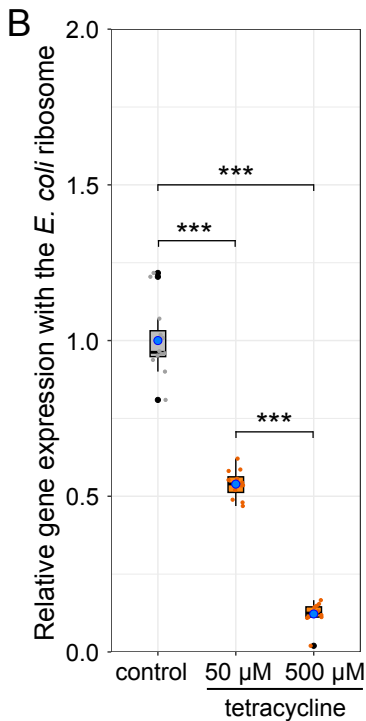

Supplement: S2 Fig — (A) Relative gene expression with the B. subtilis ribosome. The signal intensity of synthesized sfGFP in each sample is normalized to the mean signal intensity of control samples. Jitter plots of all data points (n = 12) and box plots (gray, control; green, 50 μM or 500 μM tetracycline) showing their distributions (median, quartiles, minimum, and maximum) are indicated. Blue dots represent the mean. Asterisks indicate a statistically significant difference (***, p < 0.001, Steel-Dwass test). (B) Relative gene expression with the E. coli ribosome. The signal intensity of synthesized sfGFP in each sample is normalized to the mean signal intensity of control samples. Jitter plots of all data points (n = 12) and box plots (gray, control; orange, 50 μM or 500 μM tetracycline) showing their distributions (median, quartiles, minimum, and maximum) are indicated. Blue dots represent the mean. Asterisks indicate a statistically significant difference (***, p < 0.001, Steel-Dwass test). (PDF) [file pone.0294360.s002.pdf]
